# Identification of Novel BDNF-Specific Corticostriatal Circuitries

**DOI:** 10.1523/ENEURO.0238-21.2023

**Published:** 2023-05-15

**Authors:** Yann Ehinger, Drishti Soneja, Khanhky Phamluong, Alexandra Salvi, Dorit Ron

**Affiliations:** Department of Neurology, University of California, San Francisco, 94143-0663 CA

**Keywords:** BDNF, circuitry, corticostriatal, dorsal striatum, mapping, PFC

## Abstract

Brain-derived neurotrophic factor (BDNF) is released from axon terminals originating in the cerebral cortex onto striatal neurons. Here, we characterized BDNF neurons in the corticostriatal circuitry. First, we used *BDNF*-Cre and Ribotag transgenic mouse lines to label BDNF-positive neurons in the cortex and detected *BDNF* expression in all the subregions of the prefrontal cortex (PFC). Next, we used a retrograde viral tracing strategy, in combination with *BDNF*-Cre knock-in mice, to map the cortical outputs of BDNF neurons in the dorsomedial and dorsolateral striatum (DMS and DLS, respectively). We found that *BDNF-*expressing neurons located in the medial prefrontal cortex (mPFC) project mainly to the DMS, and those located in the primary and secondary motor cortices (M1 and M2, respectively) and agranular insular cortex (AI) project mainly to the DLS. In contrast, *BDNF-*expressing orbitofrontal cortical (OFC) neurons differentially target the dorsal striatum (DS) depending on their mediolateral and rostrocaudal location. Specifically, the DMS is mainly innervated by the medial and ventral part of the orbitofrontal cortex (MO and VO, respectively), whereas the DLS receives projections specifically from the lateral part of the OFC (LO). Together, our study uncovers previously unknown BDNF corticostriatal circuitries. These findings could have important implications for the role of BDNF signaling in corticostriatal pathways.

## Significance Statement

Brain-derived neurotrophic factor (BDNF) is released in axons upon neuronal depolarization. Surprisingly, careful mapping of BDNF projecting neurons in the central nervous system (CNS) has not been conducted. Using retrograde viral strategies in combination with transgenic mice, we mapped out corticostriatal BDNF circuits. We found that, medial prefrontal cortex (mPFC) BDNF neurons project mainly to the dorsomedial striatum (DMS), whereas the motor cortex and insular cortex (AI) project to the dorsolateral striatum (DLS). BDNF neurons in the orbitofrontal cortical (OFC) are anatomically segregated. Whereas the DMS receives BDNF-positive projections from the VO, the DLS mainly receives BDNF-positive projections from the LO. Our findings could be important to the study of BDNF in corticostriatal circuitries.

## Introduction

Brain-derived neurotrophic factor (BDNF) is a member of the nerve growth factor family of neurotrophic factors, which is highly expressed in the CNS (Hofer et al., 1990; Yan et al., 1997; Kowiański et al., 2018). The majority of BDNF in neurons is stored in presynaptic dense core vesicles and is released upon neuronal depolarization (Dieni et al., 2012; Song et al., 2017). Once BDNF is released at axon terminals, it binds the receptor tyrosine kinase, tropomyosin-related kinase B (TrkB). Activation of TrkB stimulates extracellular regulated kinase 1/2 (ERK1/2), Protein kinase C (PKC) and/or phosphoinositide 3 kinase (PI3K) signaling cascades resulting in the initiation of transcriptional and translational machineries (Huang and Reichardt, 2003; Leal et al., 2014; Zagrebelsky et al., 2020). In the adult brain, BDNF plays a crucial role in synaptic and structural plasticity (Panja and Bramham, 2014; De Vincenti et al., 2019), as well as in learning and memory (Bekinschtein et al., 2014; Miranda et al., 2019).

BDNF is highly expressed in the cerebral cortex of both rodents and humans (Hofer et al., 1990; Timmusk et al., 1993; Conner et al., 1997). Studies in rodents suggest that BDNF in the cortex contributes to learning and memory paradigms (Miranda et al., 2019). For example, absence of BDNF in the prelimbic cortex (PrL) alters fear expression in mice indicating a role for consolidation and expression of learned fear (Choi et al., 2010). In the orbitofrontal cortical (OFC), BDNF is critical for goal-directed decision-making and in selecting actions based on their consequences (Gourley et al., 2013). In the motor cortex, BDNF contributes to motor learning (Andreska et al., 2020).

The cerebral cortex is also the major source of BDNF in the striatum (Altar et al., 1997; Baquet et al., 2004; Strand et al., 2007). BDNF released from cortical terminals, binds to, and activates its receptor, TrkB, in the striatum (Altar et al., 1997; Baydyuk and Xu, 2014). BDNF/TrkB signaling in the striatum has important cellular and behavioral roles (Besusso et al., 2013; Engeln et al., 2020). For example, TrkB signaling is required to control inhibition of locomotor behavior in enkephalin (ENK) positive medium spiny neurons (MSN; Besusso et al., 2013), and Lobo and colleagues provided data to suggest that BDNF/TrkB signaling in dopamine D1 receptor expressing (D1) MSN plays a role in stereotypy behaviors (Engeln et al., 2020).

Finally, rodent studies have suggested that BDNF in corticostriatal circuitries is linked to addiction. For example, numerous studies investigated the role of BDNF in the prefrontal cortex (PFC) in relation with cocaine use (for review, see Pitts et al., 2016). Specifically, cocaine exposure regulates BDNF signaling in the PFC and in turn BDNF influences the development and maintenance of cocaine-related behaviors (Lu et al., 2010; Gourley et al., 2013; Zhang et al., 2015; Pitts et al., 2018). In addition, activation of BDNF signaling in the dorsolateral striatum (DLS) keeps alcohol intake in moderation (Jeanblanc et al.,. 2009, 2013), whereas malfunctioning of BDNF/TrkB signaling in the corticostriatal regions promotes compulsive heavy use of alcohol and other alcohol-mediated behaviors (Logrip et al., 2009; Darcq et al., 2015, 2016; Warnault et al., 2016; Moffat et al., 2023).

As detailed above, BDNF is crucial for cortical and striatal functions, yet careful characterization of BDNF neurons in corticostriatal circuitry is lacking. Here, using a combination of transgenic mouse lines together with viral-mediated gene delivery approaches, we mapped out *BDNF*-expressing cortical neurons that project to the dorsomedial striatum (DMS) and DLS.

## Materials and Methods

### Reagents

Mouse anti-NeuN antibody (MAB377) was obtained from Millipore. Rabbit anti-VGLUT1 antibody (VGT1-3) was purchased from Mab Technologies. Chicken anti-GFP (A10262), donkey anti-mouse IgG AlexaFluor 594, anti-chicken AlexaFluor 488 and anti-rabbit IgG AlexaFluor 594 antibodies were purchased from Life Technologies. Other common reagents were from Sigma-Aldrich or Fisher Scientific.

### Animals and breeding

Male C57BL/6J mice (six to eight weeks old at time of purchase) were obtained from The Jackson Laboratory. Male *BDNF-*Cre knock-in mice, which express Cre recombinase at the endogenous *BDNF* locus, were obtained from Zach Knight, UCSF (Tan et al., 2016). Ribotag mice (ROSA26CAGGFP-L10a), which express the ribosomal subunit RPL10a fused to EGFP (EGFP-L10a) in Cre-expressing cells (Zhou et al., 2013), were purchased from The Jackson Laboratory (B6;129S4-Gt (ROSA)26Sortm9(EGFP/Rpl10a)Amc/J). Ribotag mice were crossed with *BDNF-*Cre mice allowing EGFP-L10a expression in *BDNF-*expressing cells. Mouse genotype was determined by PCR analysis of tail DNA.

Mice were individually housed on paper-chip bedding (Teklad #7084), under a reverse 12/12 h light/dark cycle (lights on 10 A.M. to 10 P.M.). Temperature and humidity were kept constant at 22 ± 2°C, and relative humidity was maintained at 50 ± 5%. Mice were allowed access to food (Teklad Global Diet #2918) and tap water *ad libitum*. All animal procedures were approved by the university’s Institutional Animal Care and Use Committee and were conducted in agreement with the Association for Assessment and Accreditation of Laboratory Animal Care.

### Virus information

Recombinant adeno-associated virus (rAAV) retrograde EF1a Nuc-flox(mCherry)-EGFP (1 × 10^12^ vg/ml), which expresses nuclear-localized mCherry by default but switches to nuclear-localized EGFP expression in the presence of Cre (Bäck et al., 2019; Addgene viral prep #112677-AAVrg), and AAV1-pCAG-FLEX-EGFP, expressing EGFP in the presence of Cre (Addgene viral prep #51502-AAV1), were purchased from Addgene.

### Stereotaxic surgery and viral infection

C57BL/6J (wild type; WT) or *BDNF*-Cre mice underwent stereotaxic surgery as described previously (Ehinger et al., 2020). Specifically, mice were anesthetized by vaporized isoflurane and were placed in a digital stereotaxic frame (David Kopf Instruments). A hole was drilled above the site of viral injection. The injector (stainless tubing, 33 gauges; Small Parts Incorporated) was slowly lowered into the target region. The injector was connected to Hamilton syringes (10 µl; 1701, Harvard Apparatus), and the infusion was controlled by an automatic pump at a rate of 0.1 µl/min (Harvard Apparatus). The injector remained in place for an additional 10 min to allow the virus to diffuse and was then slowly removed. For experiments investigating BDNF-positive efferent projections to the dorsal striatum (DS), *BDNF*-Cre animals were unilaterally infused with 0.5 µl of retro-AAV-EF1 aNuc-flox(mCherry)-EGFP targeting the DLS (anterior posterior (AP): +1.1, mediolateral (ML): ±2.3, dorsoventral (DV): −2.85, infusion at −2.8 from bregma) or the DMS (AP: +1.1, ML: ±1.2, DV: −3, infusion at −2.95 from bregma). For experiments investigating BDNF positive efferent projections from the OFC, *BDNF*-Cre animals were unilaterally infused with 0.5 µl of AAV1-pCAG-FLEX-EGFP targeting the vlOFC (AP: +2.58, ML: ±1.2, DV: −2.85, infusion at −2.8 from bregma).

### Immunohistochemistry, imaging, and quantification

Following intraperitoneal administration of euthasol (200 mg/kg), mice were transcardially perfused with phosphate buffered saline, followed by 4% paraformaldehyde (PFA), pH 7.4. Brains were quickly removed postperfusion and fixed for 24 h in 4% PFA before cryoprotection in 30% sucrose solution for 3 d at 4°C. Brains were then sectioned to 30 µm by cryostat (CM3050, Leica), collected serially and stored at −80°C. PFA-fixed sections were permeabilized and blocked in PBS containing 0.3% Triton X-100 and 5% donkey serum for 4 h at 4°C. Sections were then incubated for 18 h at 4°C on an orbital shaker with the primary antibodies anti-NeuN (1:500), anti-GFP (1:1000), or anti-VGlut1 (1:1000) diluted in 3% bovine serum albumin (BSA) in PBS. Next, sections were washed in PBS and incubated for 4 h at 4°C with Alexa Fluor 488-labeled donkey (1:500), Alexa Fluor 594-labeled donkey (1:500) antibodies in 3% BSA in PBS. After staining, sections were rinsed in PBS and were coverslipped using Prolong Gold mounting medium. Sections from rostral, rostrocaudal and caudal PFC were imaged on an Olympus Fluoview 3000 Confocal microscope (Olympus America) according to manufacture recommended filter configurations, using the same parameters across mice and images. Captured images were used to quantify the number of fluorescent cells in subregions of rostral, rostrocaudal, and caudal PFC using FIJI ImageJ (NIH; Schindelin et al., 2012). Specifically, the image of the PFC was precisely aligned to the anteroposterior (AP)-corresponding figure in the Paxinos atlas (Paxinos and Franklin, 2004), using the BigWarp interface included in FIJI (Bogovic, 2016). By placing corresponding landmarks on the image and atlas, the image and the AP figure were overlayed with precision. Next, regions of interest (ROIs) were traced on the overlayed image following the atlas delimitations using the polygon tool in FIJI. After adjusting the threshold (the same for each image), the number of positive neurons within an ROI was automatically quantified using FIJI counter plugin. To calculate the density of labeled neurons, the total number of labeled neurons in a region was divided by the surface of the region in mm^2^ using Fiji (Schindelin et al., 2012).

### Data analysis

GraphPad Prism 9 was used for statistical analysis. D’Agostino–Pearson normality test was used to verify the normal distribution of variables. Data were analyzed using one-way or two-way ANOVA where appropriate. One-way ANOVA was followed by Tukey’s multiple comparisons test when appropriate. For two-way ANOVAs, significant main effects or interactions were calculated, followed by Sidak’s multiple comparisons test; *p* value cutoff for statistical significance was set to 0.05.

## Results

### Experimental strategy for evaluating *BDNF*-expressing neurons distribution in the PFC

**Figure 1.**
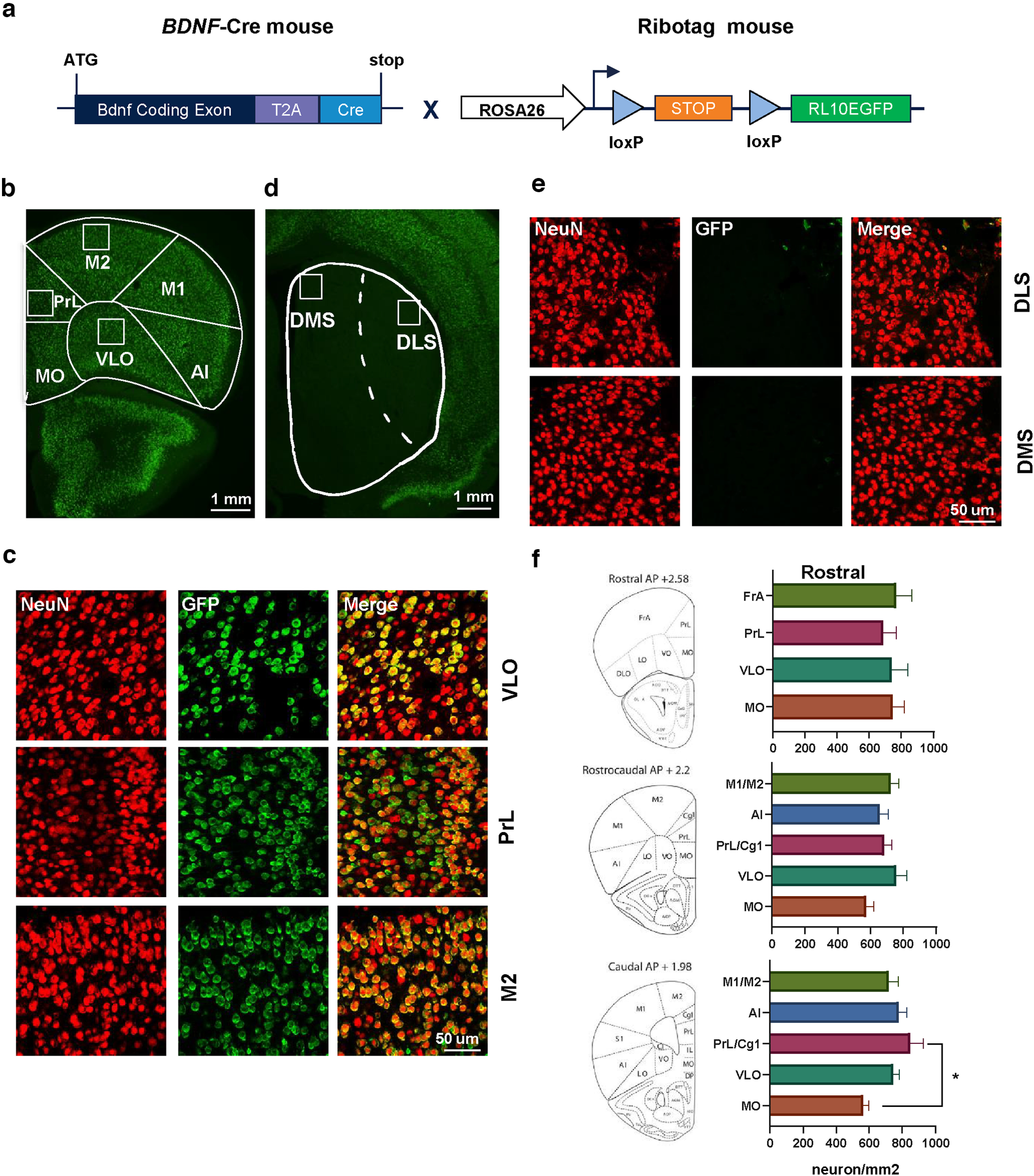
BDNF-positive neurons are detected in the PFC. ***a***, Experimental strategy for detection of *BDNF-*expressing neuron in the cortex. *BDNF*-Cre mice were crossed with Ribotag mice in which GFP-fused ribosomal subunit RPL10 is expressed in the presence of Cre recombinase, enabling the detection of *BDNF*-expressing neurons. ***b***, ***c***, Representative images of cortical neurons. NeuN (red) and GFP (green) co-localization shows a high number of *BDNF*-expressing neurons in the mPFC, MC and OFC. ***d***, ***e***, Representative images of the striatum. Neurons in the DMS and DLS do not express *BDNF*. ***f***, Topographical quantification of BDNF-positive neurons in the PFC along the rostrocaudal axis. One-way ANOVA followed by Tukey’s *post hoc* test, *p = 0.05, *n* = 5 mice. Scale bar is indicated on each panel. mPFC: medial prefrontal cortex, M1: primary motor cortex, M2: secondary motor cortex, PrL: prelimbic cortex, Cg1: cingulate area 1, OFC: orbitofrontal cortex, VLO: ventrolateral OFC, MO: medial OFC, DMS: dorsomedial striatum, DLS: dorsolateral striatum, FrA: frontal association cortex.

The cerebral cortex expresses high levels of *BDNF* message (Hofer et al., 1990; Timmusk et al., 1993); however, a careful analysis of *BDNF* containing neurons in the PFC has not been conducted. Therefore, we first assessed the distribution of *BDNF* expression in cortical neurons. To do so, we used a *BDNF*-Cre transgenic mouse line allowing Cre-recombinase expression only in *BDNF*-expressing cells (Tan et al., 2016), which was crossed with a Ribotag mouse line expressing GFP-fused ribosomal subunit RPL10 in the presence of Cre-recombinase (Zhou et al., 2013; Fig. 1*a*). The presence of GFP-fused ribosomal subunit RPL10 (EGFP-L10a) enabled the visualization of *BDNF*-expressing cells.

### BDNF-positive neurons are detected in all prefrontal regions of the cortex

A large number of *BDNF-*expressing neurons were detected in the PFC, in secondary motor cortex (M2) and the PrL as well as in the ventrolateral OFC (VLO; Fig. 1*b*,*c*). In contrast, we did not detect green fluorescence in the dorsal or ventral striatum (Fig. 1*d*,*e*), which confirms previous data indicating that striatal neurons do not express *BDNF* (Baydyuk and Xu, 2014). We then quantified the number of *BDNF*-expressing neurons in the different PFC subregions and extended frontal regions [primary motor cortex (M1) and insular cortex (AI)] along the rostrocaudal axis (Fig. 1*f*). Rostral and rostrocaudal quantification revealed that all PFC subregions exhibit the same density of BDNF-positive neurons (rostral: 735.7 ± 7.5 neurons/mm^2^, rostrocaudal: 749.3 ± 11.3 neurons/mm^2^). We did not find significant differences in the number of BDNF-positive neurons between rostral subregions (one-way ANOVA, df = 3, *F*_(3,16)_ = 0.7636, *p* = 0.5309, *n* = 5) and rostrocaudal (one-way ANOVA, df = 4, *F*_(4,20)_ = 1.888, *p* = 0.152, *n* = 5). However, caudal quantification revealed a significantly lower density in BDNF-positive neurons in the medial part of the orbitofrontal cortex (MO) compared with the PrL cingulate area 1 (Cg1; one-way ANOVA, df = 4, *F*_(4,20)_ = 3.667, *p* = 0.0214; MO vs PrL/Cg1: *p* = 0.0122, *n* = 5; Fig. 1*f*).

### Experimental strategy for mapping of prefrontal BDNF-positive neurons projecting to the DS

**Figure 2.**
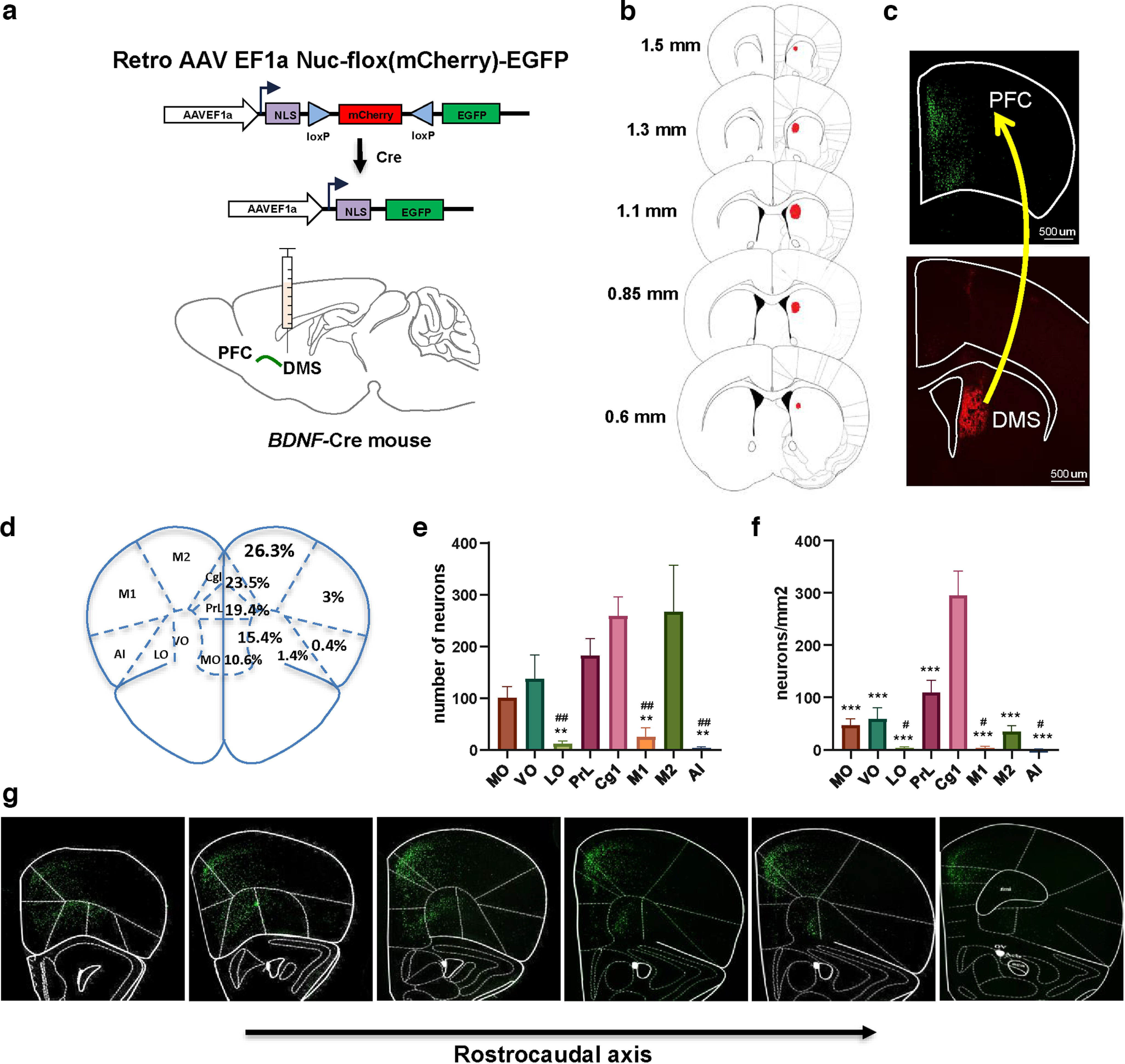
BDNF-positive neurons in the PFC project to the DMS. ***a***, Retrograde viral strategy to map *BDNF*-expressing cortical neurons projecting to the DMS. The DMS of *BDNF*-Cre mice was injected with a retrograde AAV EF1a Nuc-flox(mCherry)-EGFP viral construct enabling nuclear-localized mCherry to be expressed by default and to switch to nuclear-localized EGFP expression in the presence of Cre. ***b***, ***h***, Retro AAV EF1a Nuc-flox(mCherry)-EGFP was infused in the DMS, and BDNF-positive neurons distribution was examined in the PFC. ***b***, Rostrocaudal distribution of the viral infection site. ***c***, *BDNF*-expressing neurons were identified by EGFP-labeled nuclei and BDNF-negative neurons by mCherry nuclei. Lower panel shows BDNF-negative neurons (red) in the DMS. Upper panel shows *BDNF*-expressing neurons (green) in the PFC. ***d***, Percentage of total BDNF-positive projecting neurons per PFC subregion. ***e***, Number of BDNF-positive neurons projecting to the DMS per PFC subregion. ***f***, Density of BDNF-positive neurons projecting to the DMS per PFC subregion. ***g***, Representative images of PFC along the rostrocaudal axis. Contralateral BDNF-positive PFC projections to the DS were also quantified (Extended Data Fig. 2-1). One-way ANOVA, followed by Tukey’s *post hoc* test. #*p* < 0.05, ##*p* < 0.01 (PrL compared with other structures), **p < 0.01, ***p < 0.001 (Cg1 compared with other structures). *n* = 5 mice, scale bar is indicated on each panel. MO: medial OFC, VO: ventral OFC, LO: lateral OFC, PrL: prelimbic cortex, Cg1: cingulate area 1, M1: primary motor cortex, M2: secondary motor cortex, AI: anterior insular cortex, DMS: dorsomedial striatum.

The cerebral cortex is the major source of BDNF in the striatum (Altar et al., 1997; Baquet et al., 2004). The striatum is divided into the ventral striatum and DS (Hunnicutt et al., 2016). We focused on the DS which is further divided into the lateral (DLS) and medial (DMS) regions (Hunnicutt et al., 2016), and assessed whether *BDNF*-expressing neurons in the PFC send projections to these two parts of the DS. To do so, we used a retrograde viral strategy and infected the anterior part of the DLS or the DMS of *BDNF*-Cre mice with a retrograde AAV-Nuc-flox-(mCherry)-EGFP viral construct (Bäck et al., 2019; Fig. 2*a*), which enables mCherry expression in BDNF-negative neurons and EGFP expression in BDNF-positive neurons (Bäck et al., 2019).

10.1523/ENEURO.0238-21.2023.f2-1 Extended Data Figure 2-1 Contralateral BDNF-positive PFC projection to the DS. The DMS (***a***) or the DLS (***b***) of *BDNF-*Cre mice were injected with a retrograde AAV EF1a Nuc-flox(mCherry)-EGFP viral construct and retrogradely labeled neurons in the PFC were quantified. One-way ANOVA (*F*_(7,32)_ = 20.37; *p* < 0.0001), followed by Tukey’s *post hoc* test. ***p < 0.001, * M2 compared to other structures. *n* = 5 mice. MO: medial OFC, VO: ventral OFC, LO: lateral OFC, PrL; prelimbic cortex, Cg1: cingulate area 1, M1: primary motor cortex, M2: secondary motor cortex, AI: anterior insular cortex. Download Figure 2-1, TIF file.

### BDNF-expressing PFC neurons project to the DMS

First, we assessed whether *BDNF-*expressing cortical neurons project to the DMS by infecting the DMS of *BDNF*-Cre mice with retrograde AAV-Nuc-flox-(mCherry)-EGFP (Fig. 2*a*,*b*). As shown in Figure 2*c*, the DMS contains only cell nuclei labeled in red, corresponding to intrastriatal connections. The prefrontal regions show a high density of retrogradely EGFP-labeled neurons (Fig. 2*c*,*g*). Specifically, the mPFC including the PrL and Cg1 represents the major BDNF-positive output to the DMS, accounting for 42.9% of PFC-to-DMS projecting neurons (Fig. 2*d*,*e*). In addition, 26.3% of the BDNF-positive projections to the DMS are coming from M2 (Fig. 2*d*,*e*). The MO and ventral part of the OFC (VO) represent respectively 10.6% and 15.4% of the BDNF-positive PFC-to-DMS projecting neurons (Fig. 2*d*,*e*). Only 4.8% of total BDNF-positive neurons project from the LO, M1, and AI to the DMS (Fig. 2).

### Density of *BDNF*-expressing neurons in PFC subregions that project to the DMS

To evaluate the density of the BDNF-positive PFC neurons projecting to the DMS within each PFC subregion, we analyzed the number of retrogradely labeled neurons per mm^2^ (Fig. 2*f*). We found a significantly higher density of labeled BDNF-positive neurons in the Cg1 compared with the other regions, and in the PrL compared with LO, M1, and AI (one-way ANOVA, df = 7, *F*_(7,32)_ = 22.72, *p* < 0.0001; Cg1 vs other regions: *p* < 0.0001, PrL vs LO: *p* = 0.0206, PrL vs AI: *p* = 0.0159, PrL vs M1: *p* = 0.0204, *n* = 5; Fig. 2*f*). In addition, the BDNF-positive PFC neurons project bilaterally, but predominantly in an ipsilateral manner, and the topographic pattern of PFC *BDNF-*expressing neurons is similar in the contralateral hemisphere (Extended Data Fig. 2-1).

### *BDNF*-expressing PFC neurons project to the DLS

**Figure 3.**
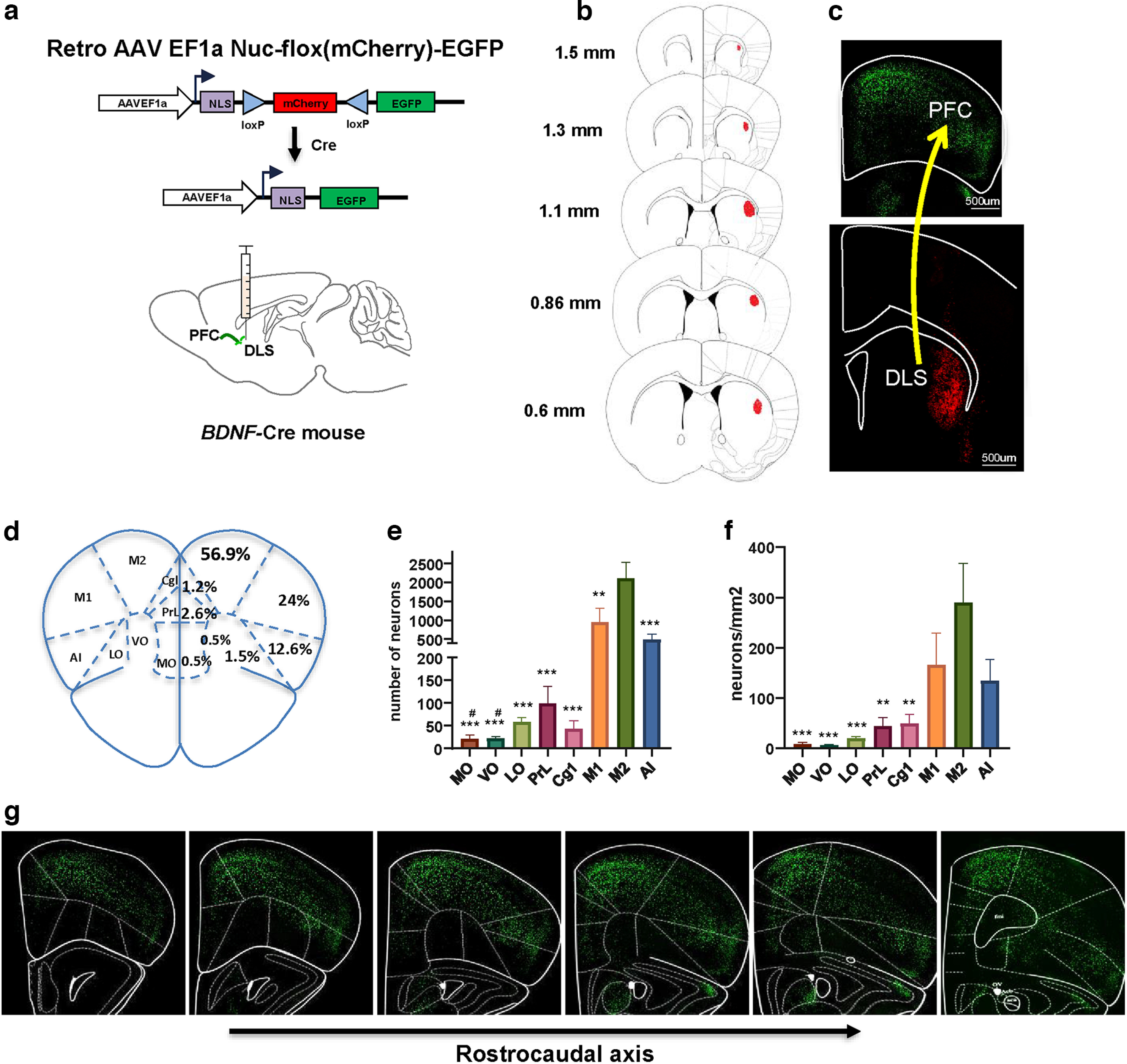
BDNF-positive neurons in the PFC project to the DLS. ***a***, Retrograde viral strategy to map *BDNF*-expressing cortical neurons projecting to the DLS. The DLS of *BDNF*-Cre mice was injected with a retrograde AAV EF1a Nuc-flox(mCherry)-EGFP viral construct enabling nuclear-localized mCherry to be expressed by default and to switch to nuclear-localized EGFP expression in the presence of Cre. ***b***, ***h***, Retro AAV EF1a Nuc-flox(mCherry)-EGFP was infused in the DLS, and BDNF-positive neurons distribution was examined in the PFC. ***b***, Rostrocaudal distribution of the viral infection site. ***c***, BDNF-positive neurons were identified by EGFP-labeled nuclei and BDNF-negative neurons by mCherry nuclei. Lower panel shows BDNF-negative neurons (red) in the DLS. Upper panel shows BDNF-positive neurons (green) in the PFC. ***d***, Percentage of total BDNF-positive projecting neurons per PFC subregion. ***e***, Number of BDNF-positive neurons projecting to the DLS per PFC subregion. ***f***, Density of BDNF-positive neurons projecting to the DLS per PFC subregion. ***g***, Representative images of PFC along the rostrocaudal axis. One-way ANOVA by Tukey’s *post hoc* test. *p < 0.05, **p < 0.01, ***p < 0.001, M2 compared with other structures. #*p* < 0.05, M1 compared with other structures. *n* = 5 mice, scale bar is indicated on each panel. VO: ventral OFC, LO: lateral OFC, PrL: prelimbic cortex, Cg1: cingulate area 1, M1: primary motor cortex, M2: secondary motor cortex, AI: anterior insular cortex, DLS: dorsolateral striatum.

Next, we assessed whether *BDNF*-expressing cortical neurons project to the DLS by infecting the DLS of *BDNF*-Cre mice with retrograde AAV-Nuc-flox-(mCherry)-EGFP (Fig. 3*a*,*b*). Similar to the DMS, the DLS contained only red cell nuclei, corresponding to intrastriatal connections (Fig. 3*c*). Absence of EGFP-labeled neurons indicated once more a lack of *BDNF* message in the DLS. However, we found a high density of retrogradely EGFP-labeled neurons in the prefrontal regions (Fig. 3*c*,*g*). Specifically, the motor cortex (M1/M2), and the AI are the main regions that send BDNF-positive projection to the DLS, representing 80.9% and 12.6%, respectively, of the overall BDNF-positive projecting neurons (Fig. 3*d*,*e*). Fewer BDNF-positive neurons in the OFC (MO, VO, and LO) and mPFC (PrL and Cg1) project to the DLS, representing together only 6.4% of the overall BDNF-positive projecting neurons (Fig. 3*d*,*e*).

### Density of PFC-to-DLS BDNF-positive projecting neurons in the PFC subregions

We next analyzed the density of retrogradely labeled BDNF-positive neurons in the PFC (Fig. 3*f*). We observed that a higher number of labeled BDNF-positive neurons are located in M2 compared with the MO, VO, LO, PrL, and Cg1 (one-way ANOVA, df = 7, *F*_(7,32)_ = 6.412, *p* < 0.0001; M2 vs MO: *p* = 0.0004, M2 vs PrL: *p* = 0.0026, M2 vs VO: *p* = 0.0004, M2 vs LO: *p* = 0.0008, M2 vs Cg1: *p* = 0.0033, *n* = 5; Fig. 3*f*).

### Comparison of topographical distribution of BDNF-positive PFC to DLS and DMS projecting neurons along the rostrocaudal axis

**Figure 4.**
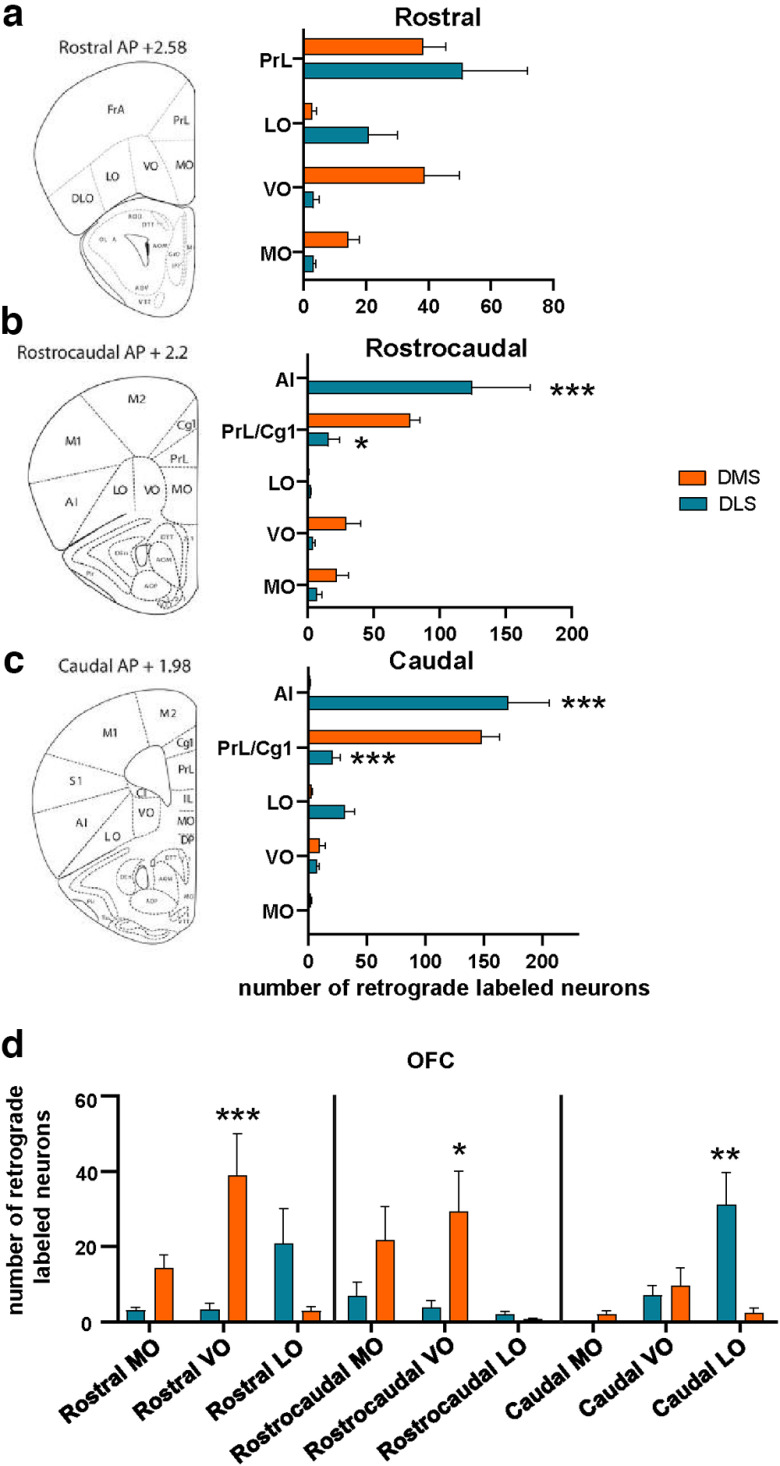
Comparison of topographical distribution of BDNF-positive PFC to DLS and DMS projecting neurons along the rostrocaudal axis. ***a–c***, Comparison of BDNF-positive PFC neurons projecting to the DMS versus DLS along the rostrocaudal axis. ***d***, Comparison of the distribution of BDNF-positive projecting neurons in the MO, VO, and LO. Two-way ANOVA, followed by Sidak’s *post hoc* test. *p < 0.05, **p < 0.01, ***p < 0.001, *n* = 5 mice. PFC: prefrontal cortex, DLS: dorsolateral striatum, DMS: dorsomedial striatum, OFC: orbitofrontal cortex, MO: medial orbitofrontal cortex, VO: ventral orbitofrontal cortex, LO: lateral orbitofrontal cortex, PrL: prelimbic cortex, Cg1: cingulate area 1, AI: anterior insular cortex.

We performed a more detailed comparison of PFC topographical patterns of DMS and DLS projecting neurons along the rostrocaudal axis (Fig. 4). There was no significant difference between the PrL, VO, LO, and MO retrogradely labeled BDNF-positive neurons at the rostral position (Fig. 4*a*). Interestingly, rostrocaudal and caudal analysis revealed a specific AI-to-DLS circuit (Fig. 4*b*,*c*). In contrast with the rostral position, retrogradely labeled BDNF*-*positive neurons in the PrL/Cg1 projecting to the DMS or DLS exhibit an opposite distribution, with fewer DLS projecting neurons and an increased number of DMS projecting neurons along the rostrocaudal axis (Fig. 4*b*,*c*). We then focused on the OFC and compared the mediolateral distribution of BDNF-positive projecting neurons along the rostrocaudal axis (Fig. 4*d*). We found that labeled *BDNF*-expressing neurons in the caudal LO are projecting significantly more to the DLS than the DMS, whereas labeled *BDNF*-expressing neurons in the rostral and rostrocaudal parts of the VO are projecting significantly more to the DMS than the DLS (two-way ANOVA; interaction effect, *F*_(8,66)_ = 6.398, *p* < 0.0001, main effect of rostrocaudal axis, *F*_(8,66)_ = 3.177, *p* = 0.0042, main effect of OFC subregion, *F*_(1,66)_ = 3.441, *p* = 0.0681, rostral VO: *p* = 0.0007, rostrocaudal VO: *p* = 0.011, caudal LO: *p* = 0.0028; *n* = 5; Fig. 4*d*).

### The DS receives inputs from BDNF-positive OFC neurons

**Figure 5.**
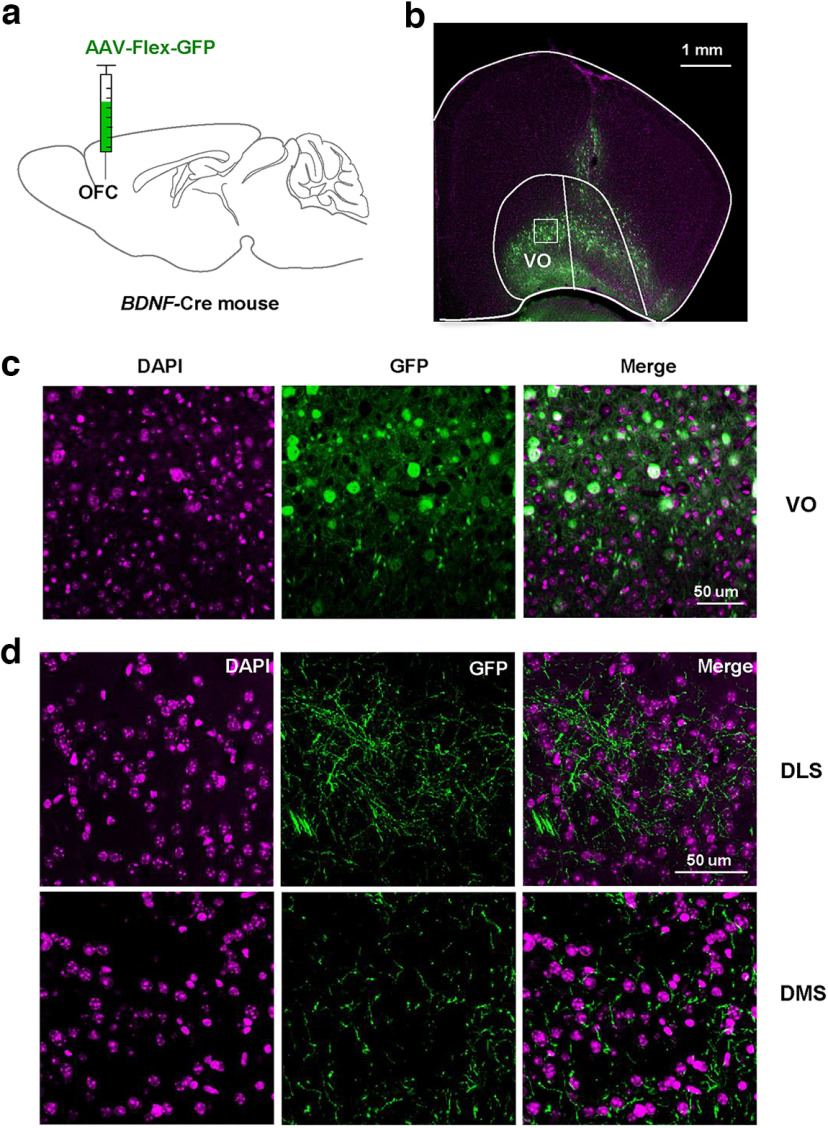
The DS receives projections from BDNF-positive OFC neurons. ***a***, ***b***, Anterograde AAV1-Flex-GFP virus was injected in the OFC of *BDNF-*Cre mice allowing visualization of projections extended by BDNF-positive OFC neurons. ***c***, ***d***, Representative images of the VO, DLS and DMS, showing GFP-positive projections localized in the DLS and in the DMS. DAPI staining is used to label nuclei. *n* = 2, scale bar is indicated on each panel. OFC: orbitofrontal cortex, DLS: dorsolateral striatum, DMS: dorsomedial striatum, DS: dorsal striatum, VO: ventral orbitofrontal cortex.

Next, we used an anterograde viral strategy to confirm the presence of a BDNF-specific circuit between the OFC and DS. To do so, we infected the OFC of *BDNF*-Cre mice with an AAV-Flex-GFP virus allowing visualization of projections extended by BDNF-positive OFC neurons (Fig. 5*a–c*). As shown in Figure 5*d*, BDNF-positive projections were localized in both the DLS and the DMS.

**Figure 6.**
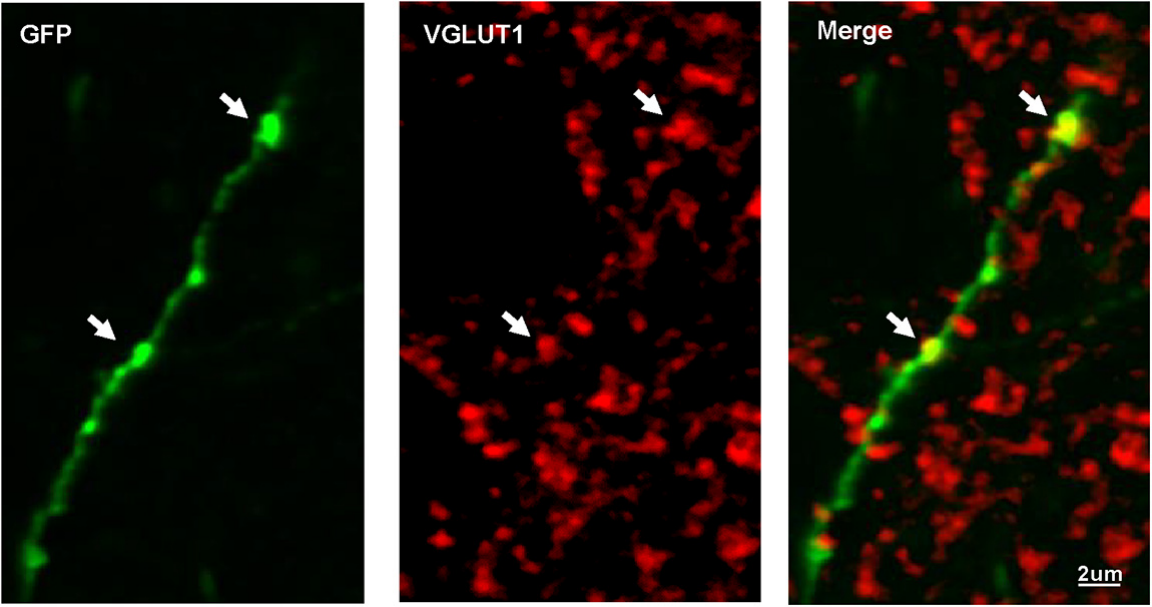
Glutamatergic BDNF-positive OFC neurons form synapses with DLS neurons. AAV-Flex-GFP virus was injected in the OFC of *BDNF-*Cre mice allowing visualization of projections in the DLS. GFP-positive projections in the DLS were stained for VGLUT1, a specific marker of cortical glutamatergic presynaptic compartment. Representative images of the DLS labeled with GFP in green and VGLUT1 in red. *n* = 2, scale bar is indicated on each panel. DLS: dorsolateral striatum, OFC: orbitofrontal cortex.

Finally, to determine whether the BDNF-positive OFC neurons form synapses with DLS neurons, we stained neurons with anti-vesicular glutamate transporter 1 (VGLUT1) antibodies labeling the cortical glutamatergic presynaptic compartment. GFP-positive projections and VGLUT1 were co-labeled in the DLS (Fig. 6), suggesting that BDNF-positive glutamatergic neurons from the OFC extend projections and form synapses with neurons in the DLS.

## Discussion

In the present study, we mapped out cortical *BDNF*-expressing neurons projecting to the DLS and DMS. We found that *BDNF*-expressing neurons located in the mPFC project mainly to the DMS, whereas the motor cortex and the AI mainly project to the DLS. Interestingly, we observed an anatomic segregation along the rostrocaudal axis of OFC BDNF-positive neurons that project to the DLS and DMS. Thus, our data define novel BDNF neural circuits connecting the PFC to the DS.

### Characterization of BDNF neurons in the PFC and the DS

In 1990, Hofer et al., used *in situ* hybridization to characterize *BDNF* message in the brain and reported that *BDNF* mRNA is abundant in the mouse cerebral cortex (Hofer et al., 1990). In contrast, BDNF protein was detected in the striatum, although *BDNF* mRNA was absent (Spires et al., 2004; Gharami et al., 2008). Altar et al., reported that BDNF protein found in the striatum is synthesized, anterogradely transported from the cell bodies located in the cerebral cortex and released presynaptically (Altar et al., 1997). Although more recent *BDNF* expression surveys have been conducted (Singer et al., 2018; Leschik et al., 2019; Wosnitzka et al., 2020), a careful mapping of *BDNF*-expressing neurons in cortical areas and their striatal projections has not been previously undertaken. Crossing a *BDNF*-Cre mouse line (Tan et al., 2016) with a Ribotag reporter line (Zhou et al., 2013) enabled labeling of *BDNF-*expressing neurons in the prefrontal cerebral cortex. Using this approach, which has superior accuracy compared with *in situ* hybridization and immunohistochemistry, we provide a definitive proof that a majority of neurons in prefrontal regions of the cortex express *BDNF*. Similar to what was previously reported (Hofer et al., 1990), we did not detect *BDNF*-expressing neurons in the striatum. Interestingly, although basal *BDNF* levels in the rodent striatum are negligible, a robust increase in *BDNF* message in the striatum is detected in response to behaviors such as voluntary alcohol intake (McGough et al., 2004; Jeanblanc et al., 2009) and cocaine administration (Liu et al., 2006), as well as in response to exercise (Marais et al., 2009) and stress (Miyanishi et al., 2021). Reconciling these potentially conflicting findings merits further investigation. One possibility is that these stimuli increase *BDNF* expression in cortical regions projecting to the striatum. Specifically, *BDNF* mRNA can be found in axon terminals (Lau et al., 2010), and recent studies show that presynaptic protein translation occurs within neuronal projections (Costa et al., 2019; Hafner et al., 2019). Thus, detectable *BDNF* in the striatum could be explained by the presence of *BDNF* mRNA within cortical neuron projections in the striatum.

### Characterization of corticostriatal BDNF circuitries

Our viral tracing strategy enabled us to explore BDNF-specific circuitries. For instance, we uncovered a specific AI-to-DLS BDNF circuit. Interestingly, Haggerty and colleagues recently found that stimulation of the AI-to-DLS circuit decreases alcohol binge drinking in male mice which in turn reshapes glutamatergic synapses in the DLS from AI inputs (Haggerty et al., 2022). In addition, we previously found that activation of BDNF/TrkB signaling in the DLS keeps alcohol intake in moderation (Jeanblanc et al., 2009, 2013). Thus, it is tempting to suggest a role for BDNF in AI-to-DLS synaptic adaptations that gate alcohol intake.

We observed that the mPFC exhibits a large number of BDNF-positive neurons projecting to the DMS. These results are in accordance with previous behavioral studies showing that the DMS receives projections from associative cortices such as the PrL (Friedman et al., 2015; Hart et al., 2018; Shipman et al., 2019; Vicente et al., 2020). We also found that a high number of BDNF-positive neurons in the motor cortex extends projections to the DLS. The motor cortex is known for innervating the DLS (Yin and Knowlton, 2006), and Andreska and colleagues recently reported that BDNF in this corticostriatal circuit is essential for motor learning (Andreska et al., 2020).

We also show herein that OFC BDNF-positive neurons project to the DS. Interestingly these projections are organized in a mediolateral manner. Specifically, the DMS receives projections from BDNF-positive neurons located in the MO and VO, whereas BDNF-positive neurons projecting to the DLS are located specifically in the LO. It is known that the OFC projects to the DS (Gremel and Costa, 2013; Gremel et al., 2016; Zimmermann et al., 2017), and previous studies showed that the DMS receives input from the MO (Gourley et al., 2016; Green et al., 2020). The DMS plays an important role for goal directed behavior (Vandaele et al., 2019), and BDNF in the MO is essential to sustain goal-sensitive action in mice (Gourley et al., 2016). Specifically, Gourley and colleagues showed that *BDNF* knock-down in the MO decreases behavioral sensitivity to reinforcer devaluation. Thus, it is likely that this BDNF circuitry and perhaps BDNF released by MO neurons within the DMS regulate goal-directed action control.

We found that the majority of LO BDNF-positive neurons project to the DLS. The functional implication of this circuitry is unknown. Gourley and colleagues reported that BDNF in both VLO and MO participates in goal-directed behavior (Gourley et al., 2013, 2016; Zimmermann et al., 2017), and Pitts and colleagues reported that inhibiting TrkB signaling in the DLS blocks habit formation (Pitts et al., 2018). In addition, mechanistic target of rapamycin complex 1 (mTORC1) in the VLO is involved in habitual alcohol seeking (Morisot et al., 2019). Putting these studies together, it is plausible that BDNF in the LO neurons projecting to the DLS plays a role in inflexible behavior.

Another possible role for BDNF in this corticostriatal circuit is to shape and modulate the synaptic output of striatal neurons. Using a two-neuron microcircuit approach in primary cortico-striatal neurons, Paraskevopoulou and colleagues showed that BDNF and glutamate co-released from cortical projections are required to modulate inhibitory synaptic transmission of striatal neurons (Paraskevopoulou et al., 2019). Therefore, co-release of BDNF with glutamate in the DLS and DMS by PFC neurons may modulate inhibitory sensitivity and dendritic morphology of striatal neurons in these PFC-DS circuits.

### BDNF-TrkB signaling in the DS

Most neurons in the striatum are MSN which are divided into two subpopulations, D1 MSN and dopamine D2 receptor (D2) expressing MSN (Gerfen et al., 1990). In the adult brain, both D1 and D2 MSN express TrkB receptors (Lobo et al., 2010). Engeln and colleagues showed that a subset of mice congenitally lacking TrkB receptor in D1-MSN in the DS exhibit repetitive circling behavior, suggesting a role of BDNF signaling in D1-MSN in this type of behavior (Engeln et al., 2020). Thus, it would be of great interest to map which dorsal striatal neuronal subtype receives inputs from cortical neuron. In addition, Nestler and colleagues provided data to suggest that activation of BDNF-TrkB signaling in D1 versus D2 MSN triggers in the nucleus accumbens (NAc) opposite effects on cocaine and morphine-dependent rewarding behaviors (Lobo et al., 2010; Koo et al., 2014). Additional studies are warranted to shed a light on the nature of cortical neurons projecting to the NAc. Furthermore, the DS is also organized into patch and matrix compartments which have distinct connectivity and genetic signature (Märtin et al., 2019). As both of patch and matrix neurons express TrkB (Costantini et al., 1999), more studies are required to decipher the contribution of BDNF/TrkB signaling in subpopulation of DS neurons.

### Comparison between our findings and the literature

Using a retrograde viral tracing, we analyzed the spatial profiles of BDNF-positive projecting neurons in the PFC. We were able to confirm that these BDNF-positive circuits exhibit the same medial–lateral gradient of corticostriatal projections first reported in rats and primates (Selemon and Goldman-Rakic, 1988; Haber, 2003; Schilman et al., 2008). Although our data are generally consistent with the excitatory corticostriatal circuits described in the literature, several points might contribute to some discrepancies. For example, Balsters and colleagues compared corticostriatal circuits between human, nonhuman primates, and mice and found significant differences in cortical projections to the anterior putamen and caudate body (Balsters et al., 2020). Many previous studies have been done in other animal models such as rats and primates (Berendse et al., 1992; Haber et al., 2006; Schilman et al., 2008; Hoover and Vertes, 2011; Mailly et al., 2013). Neuroanatomical differences exist across species and detailed explorations of similarities and differences between mice and other species are of importance. Furthermore, in contrast with previous literature (Berendse et al., 1992; Hoover and Vertes, 2011), in our comparison of the topographical distribution of BDNF-positive neurons, we observed VO-to-DMS and mPFC to-DLS BDNF-positive projecting neurons. An explanation to some of the discrepancies between corticostriatal circuit mapping studies is the experimental strategy used by us and others. Specifically, unlike the majority of studies that used anterograde strategies to map corticostriatal circuits (Berendse et al., 1992; Haber et al., 2006; Schilman et al., 2008; Hoover and Vertes, 2011; Mailly et al., 2013), we used a retrograde strategy in which a retrograde AAV was used (Tervo et al., 2016). Retrograde AAV is known to enter at the postsynaptic compartment of neurons and then be retrogradely transported to the cell body (Tervo et al., 2016). Thus, unlike an anterograde tracer which labels the entire fibers, we specifically labeled neurons projecting and forming synapses at the site of infection. However, Tervo and colleagues have provided good evidence to support the entry of AAV retrograde at axonal terminals (Tervo et al., 2016), additional studies will be necessary to fully rule out its ability to infect axons of passage. Although our anterograde tracing confirmed both the sparse and dense *BDNF*-expressing projections, anterograde tracing is not appropriate for detection of sparse projections.

Pan and colleagues reported tracing data consistent with our data using a fluorescent latex microsphere retrograde injection in mice (Pan et al., 2010). The authors reported an intense retrograde labeling in the PrL-infralimbic cortex (IL) and Cg1-M2 and no labeling in the LO-AI when injecting the retrograde tracer in the anterior DMS (aDMS). Pan and colleagues further found an intense retrograde labeling in the LO-AI and Cg1-M2 when injecting the fluorescent latex microsphere in the anterior DLS (aDLS). In addition, using a retrograde viral strategy, Green and colleagues also reported neurons located in the Cg1/PrL and IL/MO projecting to the aDMS and neurons located in the AI and M1/M2 projecting to the aDLS (Green et al., 2020).

It is important to note that our study focused specifically on the anterior part of the DS. Interestingly, Pan et al., found no retrograde labeling in the PrL, IL, MO, LO, AI, Cg1, and M2 when they injected the retrograde tracer in posterior part of the DS (pDS; Pan et al., 2010). Since the pattern of projection in the striatum differs along the AP axis (Mailly et al., 2013; Hunnicutt et al., 2016), further work is needed to decipher PFC to pDS BDNF circuits in mice. In addition, using our BDNF-positive retrograde tracing strategy, the carefully mapped corticostrial circuities were restricted to the DMS and DLS and therefore lack dorsocentral inputs. Further tracing work is needed to describe the complete mediolateral PFC-to-DS circuitries.

In summary, in this study, we mapped out *BDNF*-expressing neurons in the PFC and deciphered BDNF-specific corticostriatal circuits. Furthermore, the discovery of LO-to-DLS BDNF microcircuitry highlights the importance for deciphering the function of BDNF in the context of microcircuits composed by very localized neuronal ensembles.

## Synthesis

Reviewing Editor: Laura Bradfield, University of Technology Sydney Decisions are customarily a result of the Reviewing Editor and the peer reviewers coming together and discussing their recommendations until a consensus is reached. When revisions are invited, a fact-based synthesis statement explaining their decision and outlining what is needed to prepare a revision will be listed below. The following reviewer(s) agreed to reveal their identity: NONE.

Thanks for the revisions, the reviewers and I find the manuscript to be much improved. Nevertheless, there are a few remaining issues. Please address the specific comments from reviewers as listed below.

Reviewer #1

This is a revision of a manuscript in which authors retrogradely seeded the DMS or DLS to then label the projections from the frontal cortex that also express the neurotrophin BDNF. The majority of cortical projection neurons contain BDNF, and there is a topographical organization of innervation of the striatum, as expected based on prior investigations. A novel finding here is that about 6% of the neurons labeled following DLS seeding originated in the ventrolateral OFC + mPFC.

The revised manuscript importantly quantifies projection patterns that were previously merely descriptive. This effort revealed a paucity of projections from OFC and mPFC to DLS, which was dwarfed in number by projections from M2 and related regions. This would be expected.

Despite great improvements with several interesting quantifications, the document remains a bit perplexing to me: Overall, PL seems to have the most DLS-projecting cells (figure 3), but the authors nevertheless focus on the VLO population, finding that DLS-projecting cells are largely in the LO.

So we have a population of 6% of DLS-terminating neurons that is then further subdivided when the authors focus on those originating in the VLO. Nevertheless, the authors make a big deal of this small cell group, resulting in writing that does not match the actual data. As an example of what I mean, grand statements are made, along the lines of ‘LO BDNF+ neurons project to the DLS’ - even appearing in the Highlights. This statement implies a robust cell group and cannot be compellingly stated anyway using these methods bc only the DMS and DLS were seeded, leaving a great deal of central striatum out of the picture. (This is where most LO neurons are probably projecting.) So a more correct statement is **between the DMS and DLS,** LO neurons project to the DLS.

Another example is line 275, where the header highlights the DLS, but the data actually pertain to both DLS and DMS. And related to this experiment, here we have anterograde seeding in the OFC, and images of terminals in the striatum. However, zoomed-out images are not provided. So there’s no way for the reader to evaluate striatal subregion. This is really a weakness and missed opportunity to capture that likely central DMS innervation.

To speak plainly, it feels like the authors were really determined to find a robust LO projection to the DLS. But what was found was a quite modest projection. I recommend that the authors re-align the writing to reflect the discoveries more accurately. And relatedly, the abstract, highlights, and final paragraph of the Introduction, minimally, should all be edited to better convey the method and make clear that projections only to DMS or DLS will be detected. In other words, this isn’t really “corticostriatal mapping” - the method is more restrictive, and has an inherent bias.

Other comments:

In the new discussion paragraphs, the authors refer to “discrepancies” but don’t actually say what the discrepancy is.

A final point is that the use of “prefrontal” should be reconsidered. M1 and AI aren’t considered prefrontal.

Reviewer #2

The authors have made significant changes to the manuscript and added quantification of BDNF positive neurons in the PFC. They have also quantified projections from PFC regions to the DMS and DLS.

The information in the methods section on how these quantifications were conducted are lacking. There is no information as to how cells were quantified or how regions in the PFC were defined.

I find it a bit confusing that throughout the paper the OFC is sometimes separated into three regions but sometimes VO and LO are combined to VLO. Is there a reason for this and is there a systematic way the authors are using when referring to these different regions or combination of them?

In figure 6, the authors only look at synaptic input to the DLS even though they target both the VO and LO regions in figure 5 and show both DMS and DLS labelling. It seems a bit strange that only DLS is looked at now that some parts of the old manuscript has been removed.

There are also a few errors that might not have been updated when the manuscript was revised. E.g. figure captions descriptions are incorrect. Should it be DMS not DLS on line 223?

## Author Response

We thank the reviewers for their careful evaluation of the manuscript. To address the reviewers’ points we conducted more experiments, expended the analyses and modified the text. We believe that the manuscript is now more complete.

Comments from Reviewer 1

1. The striatal seeding strategy concerning VLO projections is flawed because DMS regions containing VLO innervation were spared (thus, the null effect is unsurprising, but should not be taken as evidence that the BDNF+ VLO neurons don’t project to the DMS, because no neurons in the VLO project to that area of DMS)

A: We have expanded experiments in new Figures 2 and 3 to assess a broader portion of the prefrontal cortex. Thus, we identified BDNF+ VLO neurons projecting to the DMS essentially located in the rostral and rostrocaudal part of the VO (new Figure 4).

2. One place where the investigation falls apart is the highly restricted striatal seeding (figure 2c), resulting a pretty incomplete picture. The finding in figure 2 showing that a viral vector infusion in the very dorsal DMS does not result in substantial labeling in the VLO is entirely unsurprising because VLO neurons don’t project to this dorsal region of the DMS. Their terminals are within the DMS, but positioned more ventrally - in other words, the authors probably just missed them. This is an excellent, focused reference: Schilman et al., 2008, Neurosci Lett, which very clearly maps these exact projections. Figure 13 of Hoover and Vertes, J Comp Neurol, 2011, might also be of interest. Notably, they do not find projections to the lateral striatum, which should be a topic of discussion. A: As mentioned above, we analyzed labeling throughout the whole rostrocaudal axis in the prefrontal cortex. In addition, we examine more carefully the striatal spread of the virus by looking at the infection in the striatum (new Figure 2b-c). We added discussion about the targeting site in the dorsal striatum and its limitation.

3. Finally, figure 4 of Mailly et al., J. Neurosci. 2013, also shows that VLO terminals ventral to the present seeding site. Note again, though, that they do not see DLS innervation. This issue should be discussed.

A: We have expanded the retrograde experiments in new Figures 2 and 3 to assess a broader portion of the prefrontal cortex and updated our discussion and conclusions to discuss the limitations in our seeding strategy. In addition, in this anterograde tracing in rats, Mailly et al. infused the PHA-L tracer in one single coordinate in the VLO, whereas we quantified the projecting neurons throughout the rostrocaudal axis of the OFC.

4. The seeding in the DLS is consistent with known regions of VLO innervation, at least per Schilman et al., 2008 (though not other groups, see above), so the discovery of BDNF+ connections there is perhaps unsurprising. The problem here, though, is that the authors have concluded that BDNF+ VLO neurons project selectively to the DLS and not DMS, based on incomplete sampling. I suspect that if the regions of the DMS where VLO projections have been documented (by many labs, including those above and those cited by the authors) were seeded here, the authors would find BDNF+ projections. If not, it would be very important to show that they could replicate prior investigations that did not use BDNF-defined projections. This exercise would validate the implication that these known VLO-to-DMS projections are for some unexpected reason BDNF-deficient.

A: The reviewer raises an important point here. We have updated the text to point out that the VLO is projecting to the DMS and DLS. We also report a mediolateral distribution within the VLO : the VO projecting mainly to the DMS and the LO to the DLS. We have also expanded our analyses and performed additional experiments, as described above, to provide a more precise 2 overview of these BDNF+ projections throughout the rostrocaudal axis. We found indeed that BDNF+ projections to the DMS are present in the more rostral part of the VO.

5. Related to this issue, in figure 4, it would be helpful to see images of the dorsal striatum at lower magnification because it does appear that anterograde tracers were detected in the DMS, and some readers will be interested to know exactly where (again, my assumption is that they are in the same regions previously reported to receive VLO innervation, but it would be good to know). And finally, in figure 5, I would include the DMS as a comparator, similar to prior figures. A: We were not able to generate the data asked by the reviewer.

6. In all, my suspicion is that a more thorough and informed survey of striatal terminal fields would look a lot like what is already know about VLO-to-striatum projections in general - that they are broad, terminating in the intermediate/medial and lateral regions of the dorsal striatum. This report would be very valuable in that it would confirm that those projection are BDNF+, which is entirely new information. I want to emphasize that adding a group in which the striatal seeding is more appropriate to investigating VLO terminals would not nullify any of the discussion points concerning the DLS. Rather, it would provide a more informed and comprehensive perspective on this issue — and likely generate more citations! A: To address the reviewer’s comment, we performed more exhaustive analysis of BDNF+ corticostriatal projections. Specifically, we quantified the distribution of the BDNF+ projecting neurons within the different PFC subregions throughout the whole rostrocaudal axis in figure 2 (PFC-to-DMS) and figure 3 (PFC-to-DLS).

7. Regardless of whether the authors opt to add this group or not, the document should be edited to be more balanced; the notion that BDNF+ VLO neurons exclusively innervate the DLS is not supported even by the current data (e.g., figure 4). A: The reviewer is correct in this assertion, and we modified the discussion so it is more aligned with the data presented.

8. The authors document mPFC projections to the DLS, a pattern that is radically different from what is known about cortico-striatal projections, and that is never discussed (the scholarship in this anatomical report is surprisingly devoid of discussion of anatomy).

A: We thank the reviewer for bringing this to our attention. We have updated the text to cite sources describing mPFC-DLS circuitry (Pan et al., 2010; Green et al., 2020) and expanded the discussion section to point out the relative novelty and potential discrepancies with other reports.

9. The second issue is the finding that the mPFC has projections to the DLS. The DLS has long been considered devoid of prefrontal input; instead, projections concentrate in the medial and intermediate zones of the dorsal striatum. For classical literature, see: Berendse et al., 1992 J Comp Neurol. For more recent investigations, see: Hunnicutt et al., 2016, eLife; Mailly et al., 2013, J.Neurosi. How do the authors reconcile these findings? Why would this technique reveal projections that have not been documented in the past? I don’t have a problem pushing against dogma, but I am having a hard time understanding how prior studies would have somehow missed BDNF+ neurons, particularly given their great density.

A: We appreciate the reviewer raising these concerns. While prior studies indeed show limited projections from the mPFC to the DLS, we do not believe that our results contradict this reporting, as only a very limited proportion of mPFC neurons project to the DLS as shown in the quantification analysis added to Figure 3 and 4. In addition, it is important to emphasize that we 3 explored and revealed BDNF-specific projections. We have updated the results and discussion sections to clarify this point.

10. Finally, virtually nothing is quantified. I suggest looking to other recent viral-mediated anatomical tracing studies for models.

A: To address the reviewer’s point, we performed additional experiments and extensive statistical analyses to strengthen our conclusions and bring this paper in line with similar recent studies.

11. The Discussion needs to be refocused on the anatomical findings reported here, relative to the large existing literature regarding these connections. While I don’t necessarily disagree with the points being made regarding BDNF or behavior, the Discussion really lacks integration with prior anatomical literature. Again, since BDNF is fairly ubiquitous and expressed at very high levels (per the authors’ figure 1), then the logical expectation is that BDNF+ cortico-striatal projections would look very similar to established patterns reported over the past few decades.

This should be explicitly noted and discussed in-depth. And if the authors are going to stick with the present conclusions, then they really need to clearly and plainly address why their data differ so significantly from existing literature.

A: We revised and refocused the discussion and the manuscript in general to contextualize our findings against a backdrop of previous, comprehensive studies of corticostriatal circuitries.

Where appropriate, as discussed above, we have walked back claims regarding DLS-exclusive projections and widespread projections from the mPFC to the DLS.

Minor comments:

1. The phrase “BDNF in corticostriatal circuitries also plays a role in addiction” should be toned down since the cited references are from rodents.

A: We updated the text to clarify that BDNF has only been shown to play a role in addiction in corticostriatal circuitries in rodents.

2. The authors use human gene nomenclature for BDNF (BDNF in italics), but it should be Bdnf (in italics) for rodent.

A: We revised BDNF nomenclature.

Comments from Reviewer 2

1. One major comment I have is that majority of the results described in the manuscript are observational. While the authors claim to have completed a careful mapping of the corticostriatal projections, this seems to be only presented in the form of one micrograph for each region at one coronal plane. There is no detail as to how the tracing experiments were analyzed. A more detailed analysis of the rostral-caudal spread of the injection and projections would have given more detail. An analysis on the density of the projections between regions would have also been useful.

A: We thank the reviewer for this critique. We added additional experiments and expanded our analyses to and have fully analyzed and presented a more thorough examination of BDNF-positive corticostriatal projections. We have expanded our analysis along the rostrocaudal axis in the cortex for each image (new Figures 2, 3 and 4). We added a description of the rostro-caudal spread within the striatum in the new Figure 2 and 3.

2. What percentage of the BDNF-positive glutamatergic neurons from the vlOFC extend presynaptic terminals with neurons in the DLS?

4

A: We performed this quantification and updated the figures and results accordingly (new Figure 3).

3. The authors claim that the projections are ipsilateral, but looking at Supplementary Figure 1, it seems there is clearly a contralateral projection. Again, perhaps if the density of the projection was quantified, then it would be easier to compare the strength of the ipsilateral and contralateral projections.

A: We performed the suggested quantification and analysis and updated the text to indicate that ipsilateral projections are denser, but contralateral projections exist as well.

4. Only photomicrographs on the ERK1/2 expression in the DMS were shown in Figure 6. What does the quantification in the DMS look like? It’s unclear how the ERK1/2 was quantified except that the authors used the Imaris software. Is there a reason why ERK1/2 cells were plotted as a % of control if the control group is also plotted? Why not show the raw numbers?

A: There was no ERK1/2 phosphorylation in the DMS and thus quantification is impossible. As Regardless, since a careful analysis required us to repeat the experiment and confirm that the results that we got are really the result of the overexpression of BDNF specifically in OFC to DLS neurons, we decided to remove Figure 6. The appropriate experiment will be conducted in the near future and will be added to a study stemming out of this study

5. In Supplementary Figure 2c, why are there two lanes for each group? Were there only two animals for each group that were analyzed? Groups sizes mentioned in Figure 6 were 5 and 4. It would be better if the expression levels were quantified. A: We removed Figure 2c, the virus is no longer used in the study.
