# Supplementary figures and images for: Identification of Novel BDNF-Specific Corticostriatal Circuitries
Source: eNeuro. 2023 May 15;10(5):ENEURO.0238-21.2023. doi: 10.1523/ENEURO.0238-21.2023 (PMC10198608; doi:10.1523/ENEURO.0238-21.2023)

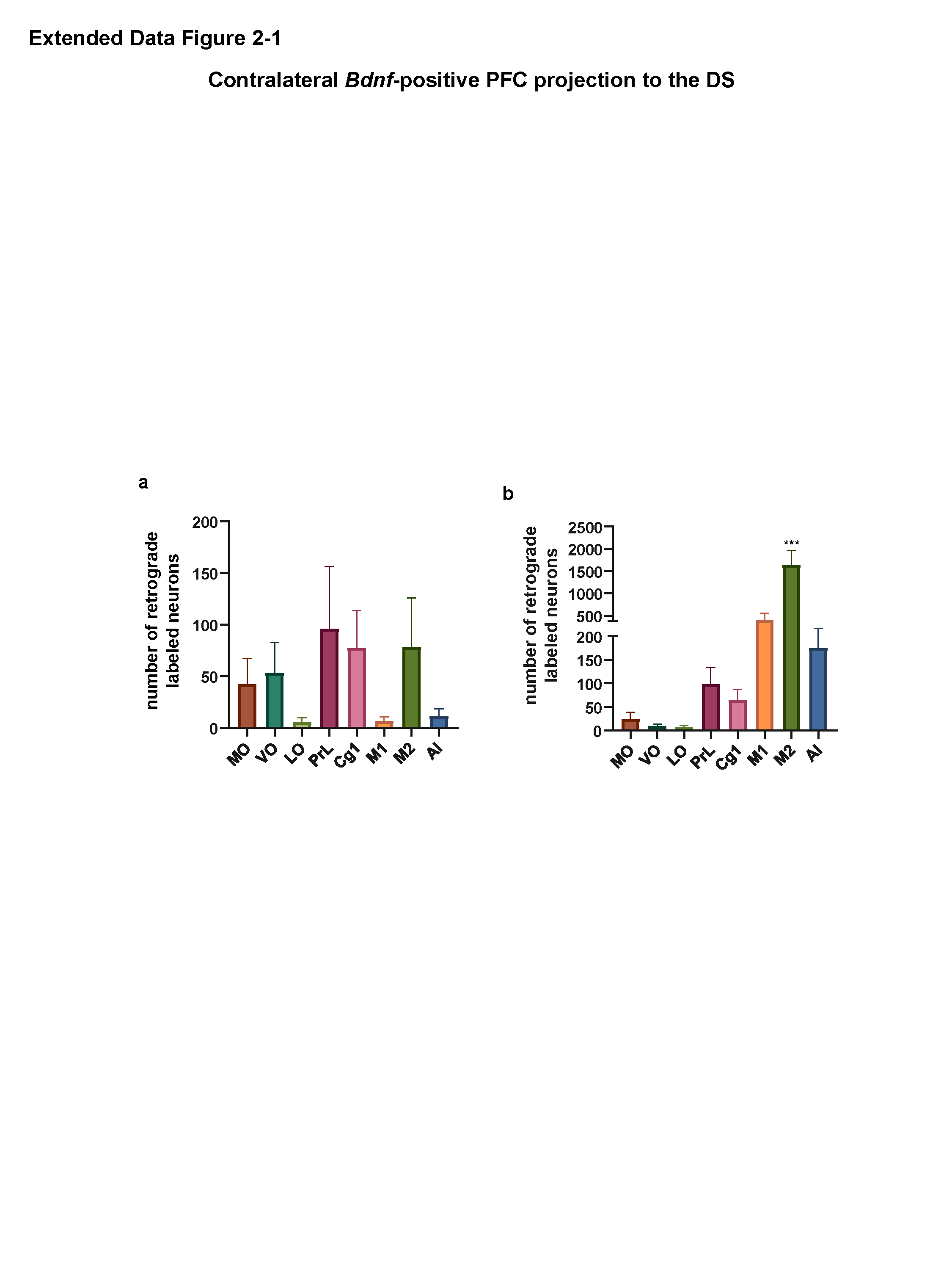

Supplement: Extended Data Figure 2-1 — Contralateral BDNF-positive PFC projection to the DS. The DMS (a) or the DLS (b) of BDNF-Cre mice were injected with a retrograde AAV EF1a Nuc-flox(mCherry)-EGFP viral construct and retrogradely labeled neurons in the PFC were quantified. One-way ANOVA (F(7,32) = 20.37; p < 0.0001), followed by Tukey’s post hoc test. ***p < 0.001, * M2 compared to other structures. n = 5 mice. MO: medial OFC, VO: ventral OFC, LO: lateral OFC, PrL; prelimbic cortex, Cg1: cingulate area 1, M1: primary motor cortex, M2: secondary motor cortex, AI: anterior insular cortex. Download Figure 2-1, TIF file. [file enu-eN-NWR-0238-21-s01.tif]
